# Exploring health workforce regulation practices and gaps in Ethiopia: a national cross-sectional study

**DOI:** 10.1186/s41256-019-0127-x

**Published:** 2019-12-11

**Authors:** Daniel Dejene, Tegbar Yigzaw, Samuel Mengistu, Firew Ayalew, Manuel Kahsaye, Damtew Woldemariam

**Affiliations:** 1 Jhpiego, Kirkos Subcity, PO Box 2881, Code 1250 Addis Ababa, Ethiopia; 2 Individual consultant, Addis Ababa, Ethiopia

**Keywords:** Health professional regulation, Registration, Licensing, Ethics, CPD, SOP, Ethiopia

## Abstract

**Background:**

Health workforce regulation plays key roles in ensuring the availability of competent health workers and improving performance of the health system. In 2010, Ethiopia established a national authority aiming to ensure competence and ethics of health professionals. Subsequently, subnational regulators were established and regulatory frameworks were developed. Although there were anecdotal reports of implementation gaps, there was lack of empirical evidence to corroborate the reports. We conducted a national study to explore health professional regulation practices and gaps focusing on registration, licensing, ethics, scope of practice, and continuing professional development.

**Methods:**

We conducted a mixed methods cross-sectional survey using structured interview with a national representative sample of health professionals and key informant interviews with health regulators and managers. We used two stage stratified cluster sampling to select health professionals. The quantitative data were subjected to descriptive and multivariable logistic regression analysis. We conducted thematic analysis of the qualitative data.

**Results:**

We interviewed 554 health professionals in the quantitative survey. And 31 key informants participated in the qualitative part. Nearly one third of the respondents (32.5%) were not registered. Many of them (72.8%) did not renew their licenses. About one fifth of them (19.7%) did nothing against ethical breaches encountered during their clinical practices. Significant of them ever practiced beyond their scope limits (22.0%); and didn’t engage in CPD in the past 1 year (40.8%). Majority of them (97.8%) never identified their own CPD needs. Health regulators and managers stressed that regulatory bodies had shortage of skilled staff, budget and infrastructure to enforce regulation. Regulatory frameworks were not fully implemented.

**Conclusions:**

Health professionals were not regulated well due to limited capacity of regulators. This might have affected quality of patient care. To ensure effective implementation of health professional regulation, legislations should be translated into actions. Draft guidelines, directives and tools should be finalized and endorsed. Capacity of the regulators and health facilities needs to be built. Reinstituting health professionals’ council and regulation enforcement strategies require attention. Future studies are recommended for assessing effects and costs of weak regulation.

## Background

Capacity to advance health services and meet people’s healthcare needs in a country is determined by the availability of a highly performing health workforce. That is why the health workforce is considered as one of the six essential building blocks of a health system, and; a competent and motivated workforce is crucial to achieve national and global health development goals [1–3].

Health workforce regulation plays key roles in ensuring the availability of competent, responsive and productive human resources for health and improving performance of the health system. Through effective regulation, arrangements are put in place to protect the public and ensure that high standards of healthcare are maintained [4]. Regulation prevents and manages harm on patients that might happen because of incompetence and malpractice [5, 6]. It builds mutual trust in quality of care among health professionals, patients and stakeholders [7]. Market failure, misuse of resources and economic inefficiency are likely to happen in a healthcare industry where there is no effective regulation [8].

Ethiopia has made impressive progress in improving health access and outcomes [9]. The recent increment in health workforce density and distribution has contributed for these successes [10]. However, unsatisfactory performance of health professionals has remained a major gap [11], partly due to lack of robust healthcare regulation [9]. As the result of this and other factors, assuring quality of healthcare has remained elusive for the country.

In 2010, the Government of Ethiopia established the Food, Medicine, and Healthcare Administration and Control Authority (FMHACA) with the mandate to protect population health by ensuring the competence and ethics of health professionals, the standards of health institutions, the safety and quality of food, the safety, efficacy, quality and proper use of medicines, and hygiene and environmental health protection. The FMHACA incorporated globally recommended regulatory frameworks for optimizing performance of the health workforce in its policy statements [10, 12, 13]. The FMHACA established 11 subnational branch offices, and delegated some regulatory duties and powers to the branch offices where it found it necessary. The FMHACA and its branches have been managing health professional registration and licensing, scope of practice (SOP), ethics and continuing professional development (CPD). Health facilities have also supported the health professional regulation practices through creating CPD opportunities, supporting healthcare ethics review system; and applying enforcement measures to strengthen regulation. Accreditation and certification examination are managed by other agencies.

Although Ethiopia adopted various health professional regulatory frameworks, their implementation has fallen behind [10, 14]. Ratified proclamations on regulation and the health professional council have not been fully enacted. Directives and guidelines on registration and licensing, CPD, SOP and ethics have not been finalized, officially endorsed or implemented. Despite issuing a CPD directive, health professionals are not asked to show evidence that they meet the requirements when they renew their licenses. There are anecdotal reports of unregistered and unlicensed practitioners, out of scope practice and ethical breaches. The health professional regulators in Ethiopia like those in other developing countries lack the necessary capacity, resources and autonomy to respond to the increasing public pressure for patient safety and redrawing professional boundaries [15–17].

However, there is a dearth of empirical evidence on gaps of the health professional regulation practices and capacity of the regulators in Ethiopia which is necessary to inform the health systems strengthening interventions. Such evidence will also expand the global knowledge base on regulation and inform regulatory practices in other low and middle income countries having similar challenges. Hence, we conducted a national study to explore health professional regulation practices and gaps focusing on registration, licensing, ethics, SOP, and CPD.

## Methods

### Study design and sample

We conducted a mixed methods cross-sectional study in March 2015. The quantitative survey was done with a nationally representative sample of health professionals working in government health facilities. The study targeted seven major clinical cadres; namely, medical doctors (including specialist doctors), health officers, nurses, midwives, anesthetists, medical laboratory professionals, and pharmacy professionals. In Ethiopia, health officers are health cadres trained for 4 years to provide clinical and public health services in rural hospitals and health centers where medical doctors are in short supply. Anesthetists are non-physician anesthesia providers with diploma or bachelor’s degree level training. According to a 2014 unpublished report from the Federal Ministry of Health (FMOH), there were 73,514 health professionals working in 2782 government health facilities (122 hospitals and 2660 health centers) in Ethiopia. We calculated a sample size of 508 health professionals using a single population proportion formula with assumptions of 95% level of confidence, proportion of 50% (since there was no prior study to estimate the proportion), design effect of 1.2 (since there was no prior study to estimate the design effect), relative error of 10%, and anticipated non-response rate of 10%. According to health facility staffing standards, the seven target health professional categories were expected to serve in hospitals but five of them (health officers, midwives, nurses, pharmacy professionals and medical laboratory professionals) were also assigned in health centers. Hence, 102 health facilities were required to fulfill the sample size taking the minimum five professional categories serving at each facility (508/5 = ~ 102). We used a power allocation technique to break down the 102 sample facilities into 22 hospitals and 80 health centers. We then proportionally allocated health facilities to the regional states.

We used a two stage stratified cluster sampling procedure to select health facilities and the target health professionals. Lists of the government health facilities in each regional state were used as sampling frames. Data collectors applied a lottery method to randomly select hospitals and health centers from the lists. Study participants were selected from lists of health professionals at each facility using the lottery method making sure that all the targeted professional categories were represented. Thus, the five professional types from each health center and the seven professional types from each hospital were included.

In situations where the selected facility was not functional or did not have the targeted professionals, it was replaced by a nearby facility. Health professionals who were full time employees and had a minimum of 6 month work experience were invited for participation. The rationale was that health professionals with fewer than 6 months at the job could not have adequate opportunity to experience regulation schemes and provide valid judgment about the health professional regulation practices and gaps.

For the qualitative part, the target populations were health regulators at FMHACA and its regional branches, and health managers at FMOH, regional health bureaus (RHBs), and selected public hospitals. We believed that the regulators and managers in these organizations possessed adequate knowledge of the implementation status and gaps of regulation. Using purposeful sampling method, we selected 24 managers and regulators for key informant interviews (KII): one from each of the 11 RHBs, 11 regional regulatory branches, the FMOH, and the FMHACA. In addition, we selected directors from 11 hospitals, one hospital from each region. The hospitals in the regions were selected based on convenience. The inclusion criteria for regulators and managers were being full time employees and having a minimum of 6 month work experience. Similarly, we thought that managers with less work experience could not give valid assessment about the health professional regulatory practices and gaps.

### Data collection

We developed two data collection tools using relevant national directives, guidelines and health workforce survey instruments [18]. The first tool was a structured questionnaire for the quantitative survey. It had 72 variables which were devoted to explore experiences and perceptions of health professionals on: registration (a process whereby a health professional data are recorded and assigned to a profession with its appropriate nomenclature), licensing (a process whereby a health professional data are recorded and assigned to a profession to its appropriate nomenclature, and a license to practice were provided to health professionals in his/her domain for 5 years), CPD (any educational activity to enhance knowledge and skills of health professionals), SOP (a list of procedures, actions and processes an individual is permitted to perform based on specific education, experiences and demonstrated competences), and ethics review system (system to protect the safety, rights, and well-being of patients and to promote ethical health care). The second tool was a key informant interview guide intended to understand process, performance and capability of the regulatory system. Four regulation experts from the FMHACA validated the tools. We pilot tested and improved the tools. Data collection was carried out by a research consultancy firm. The firm deployed 12 data collectors, each with a master’s degree and relevant work experiences. Prior to deployment, we trained the data collectors on the data collection procedures, tools and ethics. The data collectors conducted interviews with study participants in a private room. Aiming to ensure confidentiality, data on study participants’ names and their facilities were not collected. The key informant interviews were audio-recorded after getting consent from each interviewee. The data collectors were closely supported by 12 supervisors. Errors and omissions found during the data collection were corrected timely.

### Data management and analysis

For the quantitative data, questionnaires were checked for completeness and consistency of responses. The data were entered into Epi Info version 3.5.1 and exported to STATA Version 13 [19, 20]. Data cleaning was performed using both the Epi Info and STATA. We conducted data analysis using descriptive statistics and logistic regression. The logistic regression analysis was performed to look into the association between socio-demographic variables (sex, age, facility type, type of profession, level of education and work experience) and the two main outcome variables (practice beyond scope limits and participation in CPD). Multivariable logistic regression analysis was done only for variables with *p*-value less than or equal to 0.25 during binary logistic regression. We calculated 95% confidence intervals and used a p-value of less than 0.05 to determine statistical significance. Regarding qualitative data analysis, interviews were transcribed verbatim into local language (Amharic) and translated into English. The field notes were included in the transcripts. Transcription and translation of the interviews were conducted on the same day to avoid the loss of details. All transcripts were cross-checked with the audio files and the translations for consistency, correctness and completeness. Errors in the translation were corrected by referring to the Amharic versions and the audio files. We then conducted thematic analysis for the purpose of classification, summarization, and tabulation. Open Code 3.6 software was used to analyze qualitative data [21].

### Ethical Consideration

We obtained ethical clearance from the Institutional Review Board of Johns Hopkins University (JHU) Bloomberg School of Public Health. Since this research was supported by Jhpiego; an affiliate of JHU, it was a requirement for Jhpiego to obtain IRB clearance from the University. The Federal Ministry of Health and regional health bureaus also approved the study protocol and provided support letters to conduct the study. The study team members met with managers of target institutions to explain the purpose of the study and processes of data collection. Data collectors obtained informed oral consent from each study participant before data collection.

## Results

### Characteristics of study participants

A total of 554 health professionals participated in the quantitative part of the study. The response rate was 100%. Most of the study participants were sampled from health centers (72.2%). Majority of them were males (52.5%), under 30 years of age (69.1%), had non-university diploma level vocational training (52.6%), and less than 5 years of work experience (55.6%) (Table 1).

**Table 1:** Socio-demographic characteristics of study participants, exploring health workforce regulation practices and gaps, Ethiopia, 2015 (*N* = 554)

Socio-demographic Characteristics	No. of participants (%)
Age in years	
20–29	383 (69.1)
30–39	120 (21.7)
40 and above	51 (9.2)
Sex	
Male	291 (52.5)
Female	263 (47.5)
Type of Health Facility	
Hospital	154 (27.8)
Health Center	400 (72.2)
work experience in Years	
6 months to 2 Years	108 (19.5)
2 to 5 years	200 (36.1)
More than 5 years	246 (44.4)
Current profession	
Medical Doctor	22 (4.0)
Health Officer	102 (18.4)
Midwife	102 (18.4)
Pharmacy	102 (18.4)
Anesthetist	22 (4.0)
Nurse	102 (18.4)
Medical laboratory	102(18.4)
Education level	
Vocational certificate	291 (52.6)
First degree	254 (45.8)
Postgraduate degree /specialization	9 (1.6)

The qualitative part interviewed 31 of the 35 planned key informant interview (KII) participants. Four KIIs were not done because of the busy schedules of regulators and managers.

### Registration and licensing of health professionals

Out of the 554 health professionals who participated in the quantitative survey, 374 (67.5%) said they were registered for their current profession. Out of the 246 participants who had practiced for more than 5 years and hence were expected to renew their licenses as per local regulations, only 67 (27.2%) actually renewed their licenses. Moreover, of the 67 respondents who said they had renewed license, 18 (26.8%) reported that they were not asked for any evidence of fitness for practice (Table 2).

**Table 2:** Adherence to regulation requirements, exploring health workforce regulation practices and gaps, Ethiopia, 2015 (*N* = 554)

Variables	No of Participants (%)
Registered for current profession	
Yes	374(67.5)
No	180 (32.5)
Renewed professional license (*n* = 246)	
Yes	67 (27.2)
No	179 (72.8)
Evidence for professional licensure^a^	
Certificate of any in-service training	24 (35.8)
CPD credit hours	11 (16.4)
Evidence for fitness for practice (ethical and competence letter and medical certificate)	47 (70.1)
Not asked for any evidence for re-licensing	18 (26.8)
Reasons for requiring professional license: (*n* = 67)	
Personal interest	21 (31.3)
Required by the institution s/he is working	27 (40.3)
Because it is a rule	11 (16.4)
Others	8 (11.9)
Read ethics and code of conduct related document	
Yes	304 (54.9)
No	250 (45.1)
Actions against malpractice by colleague	
Took no action	109 (19.7)
Reported him/her to supervisor/facility manager	210 (37.9)
Gave professional feedback/advice	235(42.4)

^a^Totals are greater than 100% because a participant could have multiple responses and hence percentage is derived from multiple responses

The gaps in registration and licensing practices found in the quantitative survey were echoed in the qualitative part. Most key informants said that national and subnational regulators lacked the capacity to implement registration and licensing functions properly, with gaps in skilled human resources, budget, information technology infrastructure and human resource information system. On the health professionals’ side, limited awareness and fraudulent academic credentials were reported as critical challenges.

A manager in a regional health bureau said*, “Training of regulators and facility managers, and strengthening regulatory functions is needed. It is necessary* to c*reate awareness among health professionals on the importance, processes and expectations of registration, licensing and other regulations.”* (HM2)

A respondent at a subnational regulatory body said, *“The health workforce data are not completely entered into a database yet. [The] networking process [between national and subnational regulatory bodies] is not completed yet.”* (HR4)

A respondent from another subnational regulatory body stated, *“On average, we find seven cases of false certificate of competency examination during registration per month. It is very shocking.”* (HR9)

### Ethics for health professionals

Of the 554 health professionals, 250 (45.1%) said that they never read any document on health ethics and code of conduct in their entire career. In addition, 109 participants (19.7%) said they did not take any action upon encountering an ethical breach be it giving feedback or reporting to a supervisor (Table 2).

All key informants also reported that there was no a strong and systematic ethics review system at national, regional and facility levels including structures, competent staff and national standards and guidelines. On the contrary, some regional regulators and hospitals reportedly conduct ethics review processes albeit patchy. They formed ethics committees, developed guidelines and conducted supervisory visits to health facilities. They also checked on code of conduct during revalidation. However, their processes lacked standardization and consistency. In addition, many key informants acknowledged that limited awareness among health professionals, poor engagement of professional associations, scanty local evidence and weak collaboration with stakeholders were challenges for setting up an effective ethics review system.

A manager at a subnational regulatory body stated, *“We visit health facilities annually to confirm whether proper ethics and professional code of conduct are applied by health professionals. During renewing license, our body [the subnational regulator] ensures that professionals are fit for practice through requesting support letters stating ethical behaviors from their work place.”* (HR10)

A key informant in another subnational regulatory body said*, “We monitor whether professionals are working up to the level of their scope. We have our own ethics guideline at regional level but there is no endorsed national document to ensure uniformity across regions.”* (HR2)

### Scope of Practice (SOP)

Out of the surveyed health professionals, about one-fifth of them (22%) did not have adequate knowledge about their own job descriptions. More than half of them (57.9%) were not aware of their SOPs as directed by the regulatory body. Moreover, 122 respondents (22%) admitted that they conducted unauthorized tasks (tasks beyond their scope) as some point in their career while nearly twice that number (43.3%) reported knowledge of scope breach by their colleagues. The frequently mentioned reasons for practicing beyond one’s scope were shortage of qualified staff and the need to respond to life-threatening health emergencies (Table 3).

**Table 3:** Experiences regarding Scope of Practice, exploring health workforce regulation practices and gaps, Ethiopia, 2015

Variables	No of Participants (%)
Adequate Knowledge on JD	
Yes	434 (78%)
No	120 (22%)
Adequate knowledge on legal SOP (*n* = 554)	
Yes	233 (42.1)
No	321 (57.9)
Ever seen a colleague practice beyond his/her scope(*n* = 554)	
Yes	240 (43.3)
No	314 (56.7)
Measures taken against practice beyond scope (*n* = 240)	
Congratulated him/her	50 (20.8)
Told him/her not to do it again	89 (37.1)
Reported to supervisor	15 (6.2)
Reported to regulatory body	4 (1.7)
Kept quite	79 (32.9)
Others	3 (1.3)
Ever carried tasks one is not authorized to do (*n* = 554)	
Yes	122 (22.0)
No	432 (78.0)
Reasons for carrying out tasks beyond scope (122)^a^	
Absence of qualified staff	80 (72.1)
Included in the job description	2 (1.7)
Official delegation	4 (3.3)
In case of life threating emergencies	66 (54.1)

^a^percentage is larger than 100% since one participant could have multiple responses

Nurses and midwives, respectively, were thrice and twice more likely than medical doctors to perform tasks beyond their scope (AOR = 2.9; 95% CI = 1.4–5.8); and (AOR = 2.2; 95% CI = 1.1–4.7). However, scope breach was not significantly associated with type of facility, and provider age, sex, and qualification level (Table 4).

**Table 4:** Factoring affecting scope breach, exploring health workforce regulation practices and gaps, Ethiopia, 2015

Variable (Socio-demographic characteristics)	Ever carried out tasks not authorized to perform		COR (95% CI)	AOR (95% CI)
	Yes	No		
	Number (%)	Number (%)		
Type of Health Facility				
Hospital^a^	21 (13.6)	133 (86.4)		
Health Center	101 (25.3)	299 (74.7)	2.1 (1.3–3.57)^b^	1.6 (0.9–2.9)
Sex of respondent				
Male^a^	52 (17.8)	240 (82.2)		
Female	70 (26.7)	192 (73.3)	1.7 (1.1–2.5)^b^	1.2 (0.8–1.9)
Age of respondent in years				
20–29^a^	83 (21.7)	300 (78.3)		
30–39	26 (21.5)	95 (78.5)	1.00 (0.6–1.6)	
40–49	9 (25.7)	26 (74.3)	1.3 (0.6–2.8)	
50–59	4 (26.7)	11 (73.3)	1.3 (0.4–4.2)	
Current respondent qualification/profession				
Medical Doctor^a^	0 (0.0)	22 (100)		
Health Officer	21 (20.6)	81 (79.4)	1.4 (0.7–2.9)	1.4 (0.6–3.0)
Midwife	29 (28.4)	73 (71.6)	2.1 (1.1–4.2)^b^	**2.2 (1.1–4.7)**^**b**^
Anesthetist	2 (9.1)	20 (90.9)	0.5 (0.1–2.5)	0.9 (0.1–5.0)
Nurse	36 (35.3)	66 (64.7)	2.9 (1.5–5.7)^b^	**2.9 (1.4–5.8)**^**b**^
Pharmacy	18 (17.6)	84 (82.4)	1.2 (0.6–2.4)	1.2 (0.5–2.4)
Highest level of educational attainment				
Diploma^a^	74 (25.4)	217 (74.6)		
First degree	48 (18.9)	206 (81.1)	0.6 (0.5–1.0)	

^a^Reference category

^b^Significant association

Entries with bold font and astrixes show significant association

The qualitative part also showed that practicing beyond the limits of one’s scope was more common among non-physician health professionals like nurses, midwives and health officers. According to the key informants, the root cause of non-adherence to scope was scarcity of physicians. Sometimes, the health professionals are instructed by managers to complete tasks outside of their scope.

One facility manager said, “*Some professionals are assigned to work beyond their scope of practice. This happens because of lack of qualified staff and to provide care to patients with emergency.”* (HD10)

Most key informants from regional regulator bodies said that they did not have any mechanism to ensure that health professionals complied with their scope-of-practice, as the national directive and policy have not yet been endorsed. Many of them also said they often encountered conflicts between different professional groups because of lack of a scope-of-practice directive to govern professions.

A manager from a regional health bureau said, *“There is a conflict between nurses and doctors on scope of practice. Druggists have had conflicts with pharmacists. We usually face conflict between emergency surgical officers and surgeons. This happens because of lack of legally endorsed scope of practice directive.”* (HM8)

### Continuing professional development (CPD)

Nearly six out of ten health professionals (59.2%) who participated in this study reported that they were engaged in CPD activities in the last 12 months. Non-governmental organizations (NGOs) were the main CPD providers and financiers, mentioned by 71.3 and 63.7% of respondents, respectively. Although most respondents said health professionals themselves should define their learning needs (71.8%) and choose their CPD (61.4%), virtually all respondents (97.8%) claimed that their CPD experiences were arranged by others instead of being self-initiated. However, asked for their opinion on their last CPD activities, the majority said the activities were relevant (88.3%) and helped them to improve their practice (95.0%) (Table 5).

**Table 5:** Experiences regarding CPD, exploring health workforce regulation practices and gaps, Ethiopia, 2015 (*N* = 554)

Variables	No of Participants (%)
Engagement in CPD activity in the last 12 months (*n* = 554)	
Yes	328 (59.2)
No	226 (40.8)
Provider of CPD^a^ (*n* = 328)	
NGO	234 (71.3)
Government	153 (46.7)
Universities	5 (1.5)
Source of finance for the last CPD activity (*n* = 328)	
Self	5 (1.5)
Employer	54 (16.5)
NGO	209 (63.7)
FMOH/RHB	92 (28.0)
Demand of the CPD (*n* = 320)	
Self-demand	7 (2.2)
Arranged by others and invited	313 (97.8)
Perceived Relevance of the last CPD (*n* = 554)	
Relevant	489 (88.3)
Not relevant	8 (1.4)
Has not taken any CPD	57 (10.3)
Perceived practice improvement after CPD (*n* = 497)	
Yes	472 (95.0)
No	20 (4.0)
Not sure	5 (1.0)
Reasons of undertaking CPD^a^	
Career development	465 (93.6)
Want to feel confident in my work/improve performance	339 (68.2)
It is not my request	8 (1.6)
Need for incentive/reward	9 (1.8)
Preference for CPD mechanisms (*n* = 554)	
Voluntary	483 (87.2)
Mandatory	66 (11.9)
Undecided	5 (0.9)
Perceived responsibility for choosing CPD (*n* = 554)	
Health professionals	340 (61.4)
Professional associations	139 (25.1)
FMOH/RHBs	222 (40.1)
Health facilities	125 (22.6)
Regulators	28 (5.1)
NGOs	6 (1.1)
Perceived responsibility for identifying learning needs (*n* = 554)	
Health professionals	397 (71.8)
Professional associations	139 (25.1)
FMOH/RHBs	175 (31.6)
health facilities	113 (20.4)
Regulators	9 1.6)

^a^Totals are greater than 100% because the percentage is derived from multiple responses

In a bid to understand facilitators and barriers for CPD, we asked the health professionals for their perceptions about CPD. Most respondents said undertaking CPD was important for career development (93.6%) and for improving performance (68.2%). However, 87% of respondents said CPD should have been voluntary and 61.2% were against sanctions for non-compliance. Majority of respondents suggested re-certification (69.6%), career promotion (56.1%) and pay raise (50.5%) as incentives for participation. Cost (56%), shortage of time (37.6%), lack of incentive (29.2%) and not recognizing its importance (27.1%) were the most frequently mentioned barriers for CPD implementation. (Additional file 1).

Health professionals age 40 and older were less likely to participate in CPD than health professionals less than 30 years old (AOR = 0.39, 95% CI = 0.16–0.91). Pharmacy professionals were also less likely to engage in CPD activities than medical doctors (AOR = 0.23, 95% CI = 0.08–0.72). However, there was no statistically significant association with type of facility, provider sex and qualification level (Table 6).

**Table 6:** Factoring affecting participation in CPD, exploring health workforce regulation practices and gaps, Ethiopia, March 2015

Variable (Socio demographic Characteristics)	Participation of in CPD activities in the last 12 months (*n* = 554)		Crude OR (95% CI)	Adjusted OR (95% CI)
	Yes	No		
Type of Health Facility				
Hospital^a^	79 (51.3)	75 (48.7)		
Health Center	249 (62.2)	151 (37.8)	1.57 (1.08–2.28)^b^	1.41 (0.86–2.30)
Sex				
Male^a^	164 (56.2)	28 (43.8)		
Female	164 (62.6)	98 (37.4)	1.31 (0.93–1.84)	0.90 (0.60–1.33)
Age in years				
20–29^a^	246 (64.2)	137 (35.8)		
30–39	64 (52.9)	57 (47.1)	0.63 (0.41–0.95)^b^	0.78 (0.45–1.35)
40 and above	18 (36.0)	32 (64.0)	0.31 (0.17–0.58)	0.39 (0.16–0.91) ^b^
qualification/profession				
Medical Doctor^a^	15 (68.2)	7 (31.8)		
Health Officer	55 (53.9)	47 (46.1)	0.55 (0.21–1.45)	0.57 (0.19–1.73)
Midwife	74 (72.5)	28 (27.5)	1.23 (0.46–3.34)	0.90 (0.29–2.83)
Anesthetist	8 (36.4)	14 (63.6)	0.27 (0.08–0.93)^b^	0.30 (0.08–1.08)
Nurse	74 (72.5)	28 (27.5)	1.23 (0.46–3.34)	1.21 (0.39–3.76)
Pharmacy	41 (40.2)	61 (59.8)	0.31 (0.12–0.84)^b^	0.23 (0.08–0.72)^b^
Medical Lab	61 (59.8)	41 (39.0)	0.69 (0.26–1.85)	0.56 (0.19–1.70)
Highest level of education				
Diploma	184 (63.2)	107 (36.8)		
First degree and	144 (54.9)	119 (45.1)	1.40 (0.99–1.97)	1.13 (0.69–1.87)

^a^Reference Category

^b^significant association

The qualitative part of this study found out that health facilities and RHBs were not directly involved in funding, designing and delivering CPD. The RHBs and health facilities often assessed needs for CPD, appraised staff performance to select appropriate health professionals and permitted time for CPD events. However, they lacked mechanisms to track participation of health professional in and effectiveness of CPD activities.

A manager from a hospital said, “*We do not allocate budget for health professionals to engage in CPD activities. If there is any invitation, we select using internal criteria and send professionals [for training]. Very few staff identify CPD activities on their own and we don’t have any system to track staff involvement in CPD activities.”* (HD3)

A manager from RHB stressed, *“Though direct involvement of the health facilities and RHBs in providing and sponsoring CPD was low, the RHBs and FMOH are working in close collaboration with partners and teaching institutions to avail CPD for the health professionals”.* (HM5)

## Discussion

This study identified major gaps in the regulation of health professionals in Ethiopia. One third of the surveyed health professionals practiced without registration. About three quarters of respondents who served 5 years or more did not renew their licenses on time. More than four professionals out of ten never read the ethical code of conduct for health professionals and were likely to have knowledge gaps. The majority did not know their scope limits and more than one-fifth practiced beyond their qualification and authorization at least once in the past. More than a third of them did not engage in CPD in the past 1 year. The study also found out that the national and subnational regulatory bodies had limited capacity to implement the health professional regulation practices effectively.

Features of a highly performing health professional regulator include, but are not limited to, ability to translate legislations into practices, having competent staff, resources and technology, collaboration with stakeholders, using performance review results, ensuring transparency to public, and being responsive to changes in the health system [22, 23]. However, the health professional regulators in Ethiopia lacked skilled staff, budget, and technology. Favorable legal frameworks were not fully implemented [12, 13]. Regulatory directives and guidelines were drafted but not finalized or enacted [24–26]. The health professional regulatory practices in Ethiopia rarely underwent robust review, scrutiny and reform processes to meet the changes in the health system. A national study corroborated our position that the regulators have not reformed in more than a decade time other than the 2008 business process reengineering (BPR). Aiming to improve efficiency, responsiveness and customer satisfaction, Ethiopia conducted a radical reform process of the public sector including the health profession regulation functions through the BPR. As the result, the health professional regulatory functions were decentralized. Enforcement strategies and improved work processes were introduced to strengthen capacity of the national and subnational regulators [27]. Even the 2018 annual health sector performance review did not adequately scrutinize or document scrutiny results of the health professional regulation practices [28]. Because of this, the health professional regulators might find it hard to keep their pace with ever expanding number and types of health professionals and facilities, and increasing societal expectations for a safe and quality care. Without the due reforms, the regulation of health professionals in Ethiopia might be liable to outdated practices not matched with international best practices. A policy brief from World Health Organization (WHO) emphasizes that health professionals regulation in many African countries needs to be reformed [7]. But similar challenges are reported in Australia [29]. Because of technology limitations, the health professional regulation data in Ethiopia were not automated, networked and easily accessible to the public. Updating registration and licensing data could also be a challenge for the health professional regulators. As a result, the health system cannot ensure transparency and accountability. Standards for entry into professions cannot be met and maintained. Efficiency of CPD delivery and management might have been improved, had it not been for technology limitations. Weak capacity of the health professional regulators in Ethiopia implied uptake of registration, licensing and CPD among the health professionals was not satisfactory. Malpractice involving SOP breaches and ethical lapses appeared to be high. Similar to the findings of our study, key regulatory functions were not successfully implemented due to weak health professional regulation capacity in Cambodia and other African countries [30, 31].

Ethiopia has had a state led regulation rather than self- or co-regulation model. Moreover, the 2008 BPR reform reduced roles of professional associations and elements of self-regulation. Hence, it was no surprise that we found inadequate engagement of professional associations, health facilities, training institutions, and other stakeholders in the health professional regulation practices. In addition, health professionals, health facilities, RHBs and other stakeholders did not adhere to regulation enforcement strategies developed during the BPR reform process. Hiring unregistered and unlicensed professionals, lack of ethics support system and weak CPD tracking mechanisms were not taken seriously and corrected accordingly. Though the professional associations developed ethical codes, CPD courses, and the SOP for their professions, these resources were not widely utilized. In 2002, a health professional council was established and tasked with an advisory role for the national regulatory body. But the council worked only for few years and was ineffective [24]. In Cambodia, collaboration with stakeholders was also reported as weak [30]. In contrast to the situation in Ethiopia, low and middle income countries like Egypt, Nigeria and Pakistan have had functional professional councils to guide health workforce regulation [32].

Global evidences recommend establishing stronger ethics support systems for health professionals [33, 34]. However, a lack of robust ethics support functions at all levels of the health system in Ethiopia might have contributed to low ethics awareness and high malpractice among health professionals. Our study findings showed that health professionals were not supported and monitored with proper health ethics and code conduct functions. Researches from Ethiopia and other countries also reported similar problems [35–37]. Since ethical dilemmas during clinical care are generally prevalent (Challa: Ethical dilemmas and decision making process in emergency departments of experiences of physicians in Addis Ababa, Ethiopia, unpublished), in the absence of such ethics support system, the health professionals in Ethiopia might not be able to provide safe and individualized care that satisfy patients [38]. Therefore, the Government of Ethiopia should strengthen health ethics support system through building local capacity, and delegating some functions to health facilities and professional associations.

In the ideal context, health professionals should practice under professional SOP: *competencies they were adequately trained on.* But this was not happening for a number of reasons like shortage of professionals, low healthcare access and others [39, 40]. In our study, practice beyond scope limits was common especially among nurses and midwives in rural areas where there was shortage of doctors. This might be exacerbated by the occurrence of health emergencies and poor referral linkage. Because of this, many facility managers perceived working beyond one’s SOP to be ok. The unfinished national SOP directive was one of underlying reasons for the problem. Health facilities had difficulties to develop and communicate job descriptions to healthcare providers, which might have created clarity on the scopes. Endorsing the national SOP directive would inform stakeholders to ensure professional practice within the scope limits. Health facilities should communicate updated job descriptions in line with the SOP directive and follow the implementation.

The lower CPD participation rate in our study could be due to poor awareness of the importance of CPD as reported elsehwere [41–43]. It could also be the misperception that CPD is only short term face to face training. Health professionals might not report clinical update meetings, rounds, online courses, conferences, self-study and others as CPD undertakings. In contrast, health professionals in Malawi had high CPD participation rate as they were using various CPD means [44]. To be more effective, CPD must be need based and mandatory for all professionals and linked to relicensing. This was emphasized in the Ethiopian CPD directive [24]. But, the respondents in our study rarely identified their CPD needs. Removing barriers, providing incentives and designing individualized CPD events could improve CPD participation among the health professionals. The CPD courses were more donor driven and implemented by NGOs. With the large limitation of the CPD system in the country, Ethiopia might not be able to improve performance of health professionals and patient outcomes to the desired level [45].

The huge regulatory gaps found in our study might have detrimental effects on the health system performance. Unless meaningful measures are taken to revitalize national regulatory capacity, it will be difficult to ensure patient safety and quality of care. Further studies are recommended on the effects and costs of weak regulation on the health system.

### Strengths and limitations

The fact that our study was based on a national sample of the major clinical workforce should be considered a strong point. The addition of a qualitative study to understand the regulatory challenges and weaknesses in ways that might not have been known in a quantitative survey alone is another strength. However, our study included only a negligible number of health professionals with postgraduate qualifications. We also recognize that the findings may not be generalizable to health professionals in the private sector. We acknowledge that a cross-sectional study based on self-report is susceptible to recall and social desirability biases.

## Conclusions

Despite favorable legal frameworks for health professional regulation, the implementation lagged behind. Multiple gaps in the health profession regulation practices were observed. Significant strides to translate regulation policy into actions are needed through building capacity of the national and subnational regulators with appropriate staff, resources and technology. Finalizing and implementing the regulation directives and guidelines require attention. Reinstituting health professional council and enforcement strategies would improve the regulation practices. Strengthening ethics support functions at facilities is imperative. Health professionals should be trained for the additional tasks before job assignment if there is a need for them to go outside their basic scope limits. Access to need-based CPD linked to re-licensing should be prioritized. Providing incentives and removing barriers can improve CPD uptake among health professionals. Besides, future studies are suggested for assessing the effects and costs of poor regulation on the health system.

## Supplementary information

**Additional file 1:** **Table S1.** Experiences on CPD, exploring health workforce regulation practices and gaps, Ethiopia, March 2015.

## Data Availability

The datasets used and/or analyzed during this study are available from the corresponding author up on reasonable request.

## Abbreviations

AOR: Adjusted odds rate
BPR: Business process reengineering
CDC: Center for Disease Control and Prevention
CI: Confidence interval
CPD: Continuing professional development
FMHACA: Food, Medicine, Healthcare Administration and Control Authority
FMOH: Federal Ministry of Health
HD: Hospital director
HM: Health manager
HR: Health regulator
HRH: Human resources for health
HSTP: Health Sector Transformation Plan
ICN: International Council for Nurses
IRB: Institutional Review Board
JHU: Johns Hopkins University
KII: key informant interview/s
NGOs: Non-governmental organizations
RHBs: Regional Health Bureaus
SOP: Scope of practice
WHO: World Health Organization

## References

[1] World Health Organization. Monitoring the building blocks of health systems: a handbook of indicators and their measurement strategies: The WHO press; 2010. https://www.who.int.

[2] World Health Organization. Global strategy on human resources for health: workforce 2030: The WHO Press; 2016. https://apps.who.int.

[3] Speybroeck N, Kinfu Y, Dal Poz MR, Evans DB. Reassessing the relationship between human resources for health, intervention coverage and health outcomes. Evidence and information for policy: World Health Organization; 2006. https://pdfs.semanticscholar.org/fb21/eb31a783e802b2869f291ad29c80bf26ff04.pdf.

[4] World Health Organization and Australian Health Practitioner Regulation Agency. Health workforce regulation in the Western Pacific region: The WHO Press; 2014. https://apps.who.int.

[5] McKimm J, Newton PM, Da Silva A, Campbell J, Condon R (2013). Regulation and licensing of healthcare professionals: a review of international trends and current approaches in Pacific Island countries.

[6] Barry J. Regulatory board governance toolkit. ICN regulation series: International council for nurses; 2014. https://www.icn.ch.

[7] World Health Organization. Transforming and scaling up health professional education and training. Policy brief on regulation of health professions education: The WHO press; 2013. https://apps.who.int/iris/bitstream/handle/10665/93635/9789241506502_eng.pdf;jsessionid=F9018319F24B7B1A1295B75039CD06FA?sequence=1.

[8] World Health organization. Transforming and scaling up health professionals’ education and training: World health Organization guidelines; 2013. https://www.who.int/hrh/resources/transf_scaling_hpet/en/. 26042324

[9] The Federal Democratic Republic of Ethiopia Ministry of Health. Health sector transformational plan (HSTP). 2015/16–2019/20. Ethiopia; 2015. https://www.globalfinancingfacility.org/sites/gff_new/files/Ethiopia-health-system-transformation-plan.pdf.

[10] The Federal Democratic Republic of Ethiopia Ministry of Health (2016). National human resources for health strategic plan for Ethiopia. 2016–2025.

[11] Feyisa B, Herbst CH, Lemma W, Soucat A (2012). The health workforce in Ethiopia: addressing the remaining challenges, a World Bank study.

[12] Council of ministers (2014). Regulation number 299/2013. To provide food, medicine, health care administration and control, Federal negarit gazzetta.

[13] Council of ministers, Regulation number 76/2002 (2002). Ethiopian health professions council establishment, Federal negarit gazzetta.

[14] Higher education relevance and quality agency and Federal Ministry of Health (2014). Revitalizing the quality of health profession education in Ethiopia.

[15] Mccarthy CF, Voss J, Salmon ME, Gross JM, Kelley MA, Rilley PL (2013). Nursing and midwifery regulation reform in east, central and south Africa. A survey of stakeholders. Hum Resour Health.

[16] Jafari M, Currie S, Qarani WM, Azimi MD, Manalai P, Zyaee P (2019). Challenges and facilitators to establishment of a midwifery and nursing council in Afghanistan. Midwifery.

[17] Ministry of Health and Sanitation (2016). Human resources for health Sierra Leone country profile.

[18] World Health Organization (2002). Assessment of human resources for health: survey instruments and guide to administration.

[19] Center for Disease Control (CDC) (2011). Epi Info™, a database and statistics program for public health professionals. Atlanta, GA, USA.

[20] StataCorp LP (2013). Stata Statistical Software: Release 13. College Station, TX.

[21] University of Umeå (2013). ICT Services and System Development and Division of Epidemiology and Global Health. OpenCode 3.6. Sweden.

[22] Benton DC, González-Jurado MA, Beneit-Montesinos JV (2013). Defining nurse regulation and regulatory body performance: a policy Delphi study. Int Nurs Rev.

[23] Benton DC (2017). Analysis and priorities: developing a modern, effective, regulatory framework. J Nurs Regul.

[24] Ethiopian Food, Medicine and Healthcare Administration and Control Authority (2013). Directive on continuing professional development for health professionals.

[25] Ethiopian Food, Medicine and Healthcare Administration and Control Authority (2014). Directive of registration and licensure for health professionals.

[26] Ethiopian Food, Medicine and Healthcare Administration and Control Authority (2014). Directive on health professional scope-of-practice.

[27] Solomon L (2012). Framework and enforcement strategy for health professions regulation in Ethiopia.

[28] Federal ministry of health (2016). Annual performance review report: health sector transformation plan 2015/16.

[29] Wardle JL, Sibbritt D, Broom A, Steel A, Adams J (2016). Is health practitioners’ regulation keeping pace with change in practitioner and health care landscape? An Australia perspective. Front Public Health.

[30] Clarke D, Duke J, Wuliji T, Smith A, Phuang K, San U (2016). Strengthening health professional regulation in Cambodia: a rapid assessment. Hum Resour Health.

[31] Gross JM, Kelley M, McCarthy C. The model for advancing professional nursing collaboration. Africa health professions regulation collaborative. J Nurse Regul. 2015;6(3). www.journalofnursingregulation.com.

[32] de Vries H, Sanderson P, Janta B, Rabinovich L (2009). International comparison of ten medical regulatory systems: Egypt, Germany, Greece, India, Italy, Nigeria, Pakistan, Poland, South Africa and Spain.

[33] World health organization (2015). Global health ethics key issues, global networks of WHO collaborating centers on bioethics.

[34] Rasaol D, Skovdahl K, Gifford M, Kihlgren A (2017). Clinical ethics support for health professionals: an integrative literature review. HEC Forum.

[35] Tiruneh MA, Ayele BT (2018). Practice of code of ethics and associated factors among medical doctors in Addis Ababa Ethiopia. PLoS One.

[36] Milieteig I, Onaheim K, Johansson KA BF, Dessalegn D. Ethics capacity building in low-income countries: Ethiopia as a case study. Tidsskrift for den Norske Legeforening. 2017;137(22). https://tidsskriftet.no/sites/default/files/generated_pdfs/49805-ethics-capacity-building-in-low-income-countries-ethiopia-as-a-case-study.pdf. 10.4045/tidsskr.17.0759 29181929

[37] Adhikari S, Aro AR, Paudel K, Adhikari TB (2016). Knowledge, attitude and practice of healthcare ethics among resident doctors and ward nurses in resource poor setting, Nepal. BMC Med Ethics.

[38] Ogunbanjo GA, Knapp van Bogaert D (2014). Ethics in health care, the practice of defensive medicine. S Afr Fam Pract.

[39] Federation of state medical boards (2005). Assessing scope of practice in health care delivery. critical questions in assuring public access and safety USA.

[40] Dower C, Moore J, Langelier M (2013). It is time to restructure health professionals’ scope of practice regulations to remove barriers to care: analysis and commentary. Health Aff.

[41] Assemelash D, Binega G, Kass M. Involvement of community pharmacist in continuing professional development (CPD). A baseline survey Gondor, Northwest Ethiopia. Global Health. 2018;14(1):15. https://www.ncbi.nlm.nih.gov/pubmed/29391021. 10.1186/s12992-018-0334-0 PMC5796592 29391021

[42] Wiam E, Elamrdi A, Alyafie S, Abuzaid M. Continuing professional development in radiography: practice, attitude and barriers; 2015.

[43] Feldack C, Pintye J, Jacob S, Chung MS, Middleton L (2017). Continuing professional development for medical, nursing, and midwifery cadres in Malawi, Tanzania and South Africa: A qualitative evaluation. PLoS One.

[44] Muula AS, Misiri H, Chimalizeni Y, Mpando D, Phiri C, Nyaka A (2004). Access to continued professional education among health workers in Blantyre, Malawi. Afr Health Sci.

[45] Mathers N, Mitchell C, Hunn A (2012). A study to assess the impact of continuing professional development (CPD) on doctors’ performance and patient/service outcomes for the GMC; academic unit of primary care, The University of Sheffield, Capta Health.

