# Supplementary material for: Exploring health workforce regulation practices and gaps in Ethiopia: a national cross-sectional study
Source: Glob Health Res Policy. 2019 Dec 11;4:36. doi: 10.1186/s41256-019-0127-x (PMC6905006; doi:10.1186/s41256-019-0127-x)
Supplement: Supplementary file 1 — Additional file 1: Table S1. Experiences on CPD, exploring health workforce regulation practices and gaps, Ethiopia, March 2015. [file 41256_2019_127_MOESM1_ESM.docx]

**Table: Experiences on CPD, exploring health workforce regulation practices and gaps, Ethiopia, March 2015.**

| **Variables** | **No of Participants (%)** |
| --- | --- |
| **Incentives for participating in CPD (n=554)*** |  |
| Re-certification | 385 (69.5) |
| Pay rise (increase in fees | 279 (50.4) |
| Career promotion | 310 (56.0) |
| List of Providers participating on CPD will be published | 79 (14.3) |
| **Agreement for sanction if no CPD participation (n=554)** |  |
| Yes | 177 (31.9) |
| No | 339 (61.2) |
| Neither | 38 (6.9) |
| **Type of proposed sanctions (n=177)** |  |
| Suspend right for practice | 36 (20.3) |
| Official warning | 138 (78.0) |
| Salary deduction or hold promotion | 3 (1.7) |
| **Barriers for participating in CPD (n=554)*** |  |
| Lack of incentives | 162 (29.2) |
| Shortage of time | 208 (37.5) |
| High cost | 310 (56.0) |
| Lack of awareness | 102 (18.4) |
| Not recognizing its importance | 15 (2.7) |
| **CPD was individualized for one’s learning needs (n-497)** |  |
| Yes | 484 (97.4) |
| No | 12 (2.4) |
| Not sure | 1 (0.2) |

_____________________________________________________________________________________

* Totals are greater than 100% because the percentage is derived from multiple responses
